# Anti-apoptotic BCL-2 regulation by changes in dynamics of its long unstructured loop

**DOI:** 10.1038/s42003-020-01390-6

**Published:** 2020-11-12

**Authors:** Yu-Jing Lan, Pei-Shan Yeh, Te-Yu Kao, Yuan-Chao Lo, Shih-Che Sue, Yu-Wen Chen, Dennis W. Hwang, Yun-Wei Chiang

**Affiliations:** 1 Department of Chemistry, National Tsing Hua University, Hsinchu, Taiwan; 2 Biomedical Translation Research Center, Academia Sinica, Taipei, Taiwan; 3 Institute of Bioinformatics and Structural Biology, National Tsing Hua University, Hsinchu, Taiwan; 4 Institute of Biomedical Sciences, Academia Sinica, Taipei, Taiwan

**Keywords:** Intrinsically disordered proteins, Biophysical chemistry, Structural biology

## Abstract

BCL-2, a key protein in inhibiting apoptosis, has a 65-residue-long highly flexible loop domain (FLD) located on the opposite side of its ligand-binding groove. In vivo phosphorylation of the FLD enhances the affinity of BCL-2 for pro-apoptotic ligands, and consequently anti-apoptotic activity. However, it remains unknown as to how the faraway, unstructured FLD modulates the affinity. Here we investigate the protein-ligand interactions by fluorescence techniques and monitor protein dynamics by DEER and NMR spectroscopy tools. We show that phosphomimetic mutations on the FLD lead to a reduction in structural flexibility, hence promoting ligand access to the groove. The bound pro-apoptotic ligands can be displaced by the BCL-2-selective inhibitor ABT-199 efficiently, and thus released to trigger apoptosis. We show that changes in structural flexibility on an unstructured loop can activate an allosteric protein that is otherwise structurally inactive.

## Abstract (web summary)

Lan et al. make single and triple phosphomimetic mutations in a long flexible loop domain of BCL-2 and show that these mutations reduce structural flexibility and promote ligand access to the groove. Their study indicates that changes in structural flexibility on an unstructured loop can activate an allosteric, otherwise inactive protein.

## Introduction

Anti-apoptotic BCL-2 protein is a key regulator of the apoptotic process. Due to its widespread expression in various malignancies, it is considered as a predictive biomarker or therapeutic target in the diagnosis of cancer^1–3^. BCL-2 was previously shown to inhibit cell death by preventing the binding of the BH3-only (or pro-apoptotic) proteins to the mitochondrial outer membrane (MOM). Thus, overexpression of BCL-2 can suppress MOM permeabilization (MOMP) in numerous cell death pathways, preventing the release of apoptogenic factors such as cytochrome *c*, and SMAC/DIABLO from mitochondria.

**Fig. 1:**
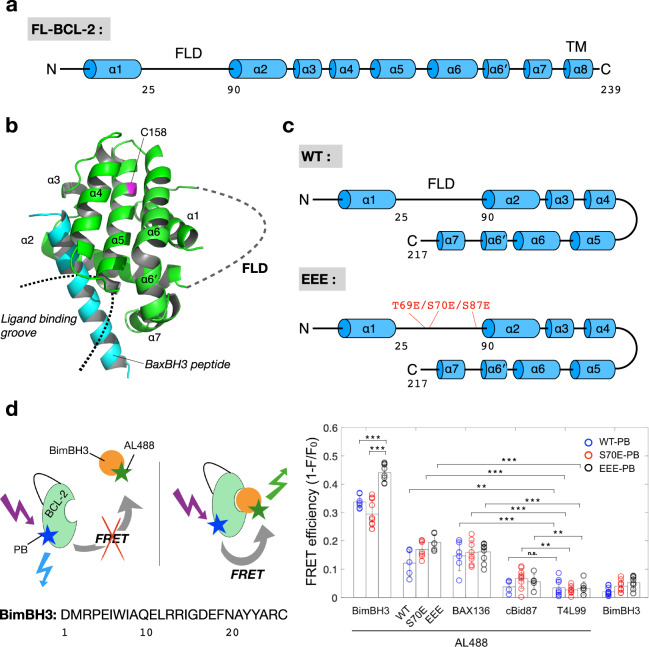
Structure and ligand-binding groove of BCL-2. **a** Cartoon representation (helices shown as cylinders) of full-length BCL-2. **b** Cartoon model of the previously reported BCL-2-ΔTM/FLD structure (PDB: 2XA0), exhibiting the ligand-binding groove of BCL-2. The only native cysteine site 158 is clearly away from the binding groove, and thus suitable for conjugating a fluorescence probe. **c** Cartoon representations of the studied WT and EEE samples, in which the only difference is the phosphomimetic mutations T69E/S70E/S87E. FLD corresponds to the sequence 25–90. **d** Association of BimBH3 and BCL-2 is examined by FRET. Sequence of BimBH3 peptide is given. BimBH3-AL488 is associated with EEE-PB more potently than with S70E-PB or WT-PB, as compared with other controls (WT-, S70E-, EEE-, BAX136-, cBid87-, T4L99-AL488). Data are shown as mean ± SD (*n* > 4).

The anti-apoptotic activity of BCL-2 can be regulated by post-translational modifications^4–8^. Several in vivo studies reported that BCL-2 phosphorylated at sites T69, S70, and S87 (or with phosphomimetic mutations, such as T69E/S70E/S87E) is enhanced to protect cells against apoptotic cell death, suggesting that the phosphorylations (or by phosphomimetic substitutions) increase the anti-apoptotic activity of BCL-2^5,9–11^. However, the three phosphorylation-related residues are in a 65-residue-long, highly flexible loop domain (denoted by FLD, herein; Fig. 1a) that is often removed for simplicity in structural studies of NMR and X-ray crystallography. Thus, the role of the FLD in the functional regulation of BCL-2 has been missing from the available structural data. It remains unclear by what mechanism the phosphorylation status of the highly flexible loop affects the activity of BCL-2. The FLD, in fact, is located on the opposite side of the known binding groove of BCL-2. The mechanism of how the far-off FLD affects the affinity of BCL-2 for apoptotic activators has been a long-standing puzzle.

BCL-2 is a tail-anchored protein; namely, it contains a C-terminal hydrophobic helix (α8) that functions as a transmembrane (TM) domain. The structure of full-length BCL-2 (FL-BCL-2, Fig. 1a) protein has not been determined, but there have been reports of various truncated BCL-2 structures (e.g., PDB codes: 2XA0, 1GJH, 4MAN), all of which lack the FLD and TM (see Supplementary Fig. 1 for a detailed comparison among the truncated structures)^12–14^. Fig. 1b shows the crystal structure 2XA0, wherein its TM helix and the FLD were truncated in the study. This crystal structure, though truncated, reveals the binding domain of BCL-2 for various (BH3-domain-based) pro-apoptotic peptides, providing valuable information for understanding the structure–function relation of BCL-2 protein. There are some similarities among anti-apoptotic proteins (such as BCL-2 and BCL-xL) in the BCL-2 protein family; they share a common motif comprising C-terminal hydrophobic-helices surrounded by several amphipathic-helices. In BCL-2, four of these amphipathic helices (α2–α5) form a hydrophobic groove serving as the binding site for pro-apoptotic activators (Fig. 1b).

It is worth noting that the binding groove is also responsible for the engagement of an orally bioavailable small molecule ABT-199 (Venetoclax)^8,13^. ABT-199 has been shown to be a BCL-2-selective inhibitor that potently inhibits the growth of BCL-2-dependent tumors. ABT-199 can displace the bound pro-apoptotic activator from the groove of BCL-2 efficiently and, consequently, cause the release of pro-apoptotic activators, promoting MOMP and apoptosis. This study will use ABT-199 to examine whether the BCL-2 mutants studied here have the same response as FL-BCL-2 to ABT-199.

The present study aims to reveal the molecular mechanism underlying the enhanced anti-apoptotic activity due to phosphorylations on the FLD using Förster resonance energy transfer (FRET), fluorescence recovery after photobleaching (FRAP), and pulsed ESR double electron–electron resonance (DEER) spectroscopic tools^15–19^. We study changes in the ligand-binding activity, anti-apoptotic capacity, and structural conformation of various recombinant BCL-2 mutants. The studied BCL-2 mutants include a TM-truncated wild-type BCL-2 (denoted as WT, herein; Fig. 1c), two phosphomimetic WT mutants, which include T69E/S70E/S87E mutant (denoted as EEE; Fig. 1c) and a less potent phosphomimetic mutant S70E, and various double-cysteine variants of mutants for spin-label ESR studies.

## Results and discussion

### Study of BCL-2 and ligand associations by FRET

The anti-apoptotic activity of BCL-2 can be evaluated by examining its affinity for apoptotic activators. This study used a 26-mer-long BimBH3-domain-based peptide (see Fig. 1d for sequence) to induce the activation of pro-apoptotic BAX protein and MOMP^18,20–22^. Interactions of several BH3-only-based peptides with pro-survival proteins have been previously reported, showing that they would vary substantially in affinity with different peptide lengths and truncated forms of the proteins^14,23–25^. To measure the interaction of this specific BimBH3 peptide with the BCL-2 mutants of the present study, we performed FRET with fluorescence probes Pacific Blue (PB) and Alexa Fluor 488 (AL488), as donor and acceptor, respectively. Our FRET results (Fig. 1d) revealed that the molecular association of BimBH3 with EEE is much stronger than those associations of BimBH3 with S70E or WT, as compared with other control experiments shown. This finding is also supported by other FRET measurements (Supplementary Fig. 2).

### In vitro cytochrome *c* release assay to evaluate the activity of BCL-2

**Fig. 2:**
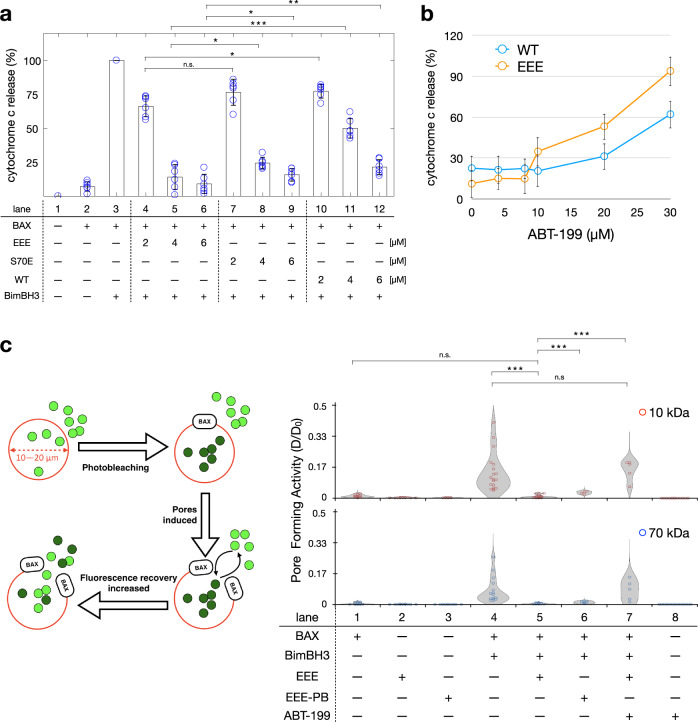
In vitro assays for evaluating BCL-2 activity. **a** Cytochrome *c* release assay with various BCL-2 mutants at varying concentrations. All of the release amounts are normalized to the result caused by the BimBH3-induced BAX activation (lane 3). Among the BCL-2 proteins studied (WT, S70E, and EEE), EEE is the most potent in inhibiting the BAX-induced MOMP. Concentrations of BAX (0.1 μM) and BimBH3 (2 μM) are fixed in all in vitro assays. Data are shown as mean ± SD (*n* = 6). **b** Cytochrome *c* release assay (as in **a**) at varying amount of ABT-199. Adding ABT-199 is useful to displace BimBH3 from the BCL-2-binding groove, thus promoting the BimBH3-induced BAX activation and MOMP. **c** FRAP experiment to measure the pore-forming activity of BAX. GUVs provide a MOM-like environment devoid of endogenous mitochondrial proteins. EEE is effective in sequestering BimBH3 and preventing BAX-induced membrane pores. ABT-199 can displace BimBH3 from EEE (lanes 5 and 7). Data are shown as mean ± SD (*n* > 20).

The strong association between EEE and BimBH3 (as shown in Fig. 1d) may directly prevent BimBH3 from activating BAX, thus leading to an enhancement in the anti-apoptotic activity of BCL-2. To verify this implication, we performed in vitro cytochrome *c* release assay using isolated mitochondria. Pro-apoptotic BimBH3 peptide was applied here as an activator to trigger BAX-induced MOMP, which causes the release of cytochrome *c* from mitochondria. BAX alone is inactive (lane 2 in Fig. 2a), as a negative control. Lane 3 (Fig. 2a) is a positive control for the cytochrome *c* release assays. In the presence of EEE (lanes 4–6), the ability of BAX to cause MOMP is largely inhibited as BimBH3 is sequestered and prevented from activating BAX. The capacity of EEE to inhibit the BAX-induced MOMP was observed clearly to increase with increasing the amount of EEE added, and it was evidently greater than those of S70E (lanes 7–9) and WT (lanes 10–12). (More experimental results in support of the observed BCL-2 activity are given in Supplementary Fig. 3.)

As ABT-199 is a BCL-2-selective inhibitor, we next examine (Fig. 2b) whether in the presence of ABT-199, BimBH3 can be released from the binding groove of BCL-2 to cause BAX-induced MOMP. The cytochrome *c* release assays were performed at varying amounts of ABT-199. The greater the amount of ABT-199 was added (Fig. 2b) to the pre-incubated solution containing BCL-2, BAX, BimBH3, and mitochondria, the greater the release of cytochrome *c* was detected. The results support that ABT-199 can displace BimBH3 from BCL-2 (either EEE or WT). The presence of ABT-199 can positively regulate the amount of free BimBH3 in the assay for cytochrome *c* release from mitochondria.

Taken together the FRET and the cytochrome *c* release assay results, it suggests that the greater affinity of BimBH3 for EEE than for S70E or WT is the major cause that leads to the enhanced anti-apoptotic activity of BCL-2. As EEE has a high affinity for BimBH3, the pro-apoptotic BimBH3 peptides, when incubated with BAX and EEE in the presence of mitochondria, are thus sequestered and prevented from inducing BAX activation and MOMP. Moreover, it suggests that the incubation of EEE with BimBH3 could render BCL-2 into a primed-to-kill state, which is sensitive to apoptosis-inducing ligands such as ABT-199.

### FRAP-GUV assay establishes the connections among BCL-2, BimBH3, ABT-199, and BAX

There are various endogenous proteins present at the MOM and they could somewhat interact with BAX or BCL-2, thus complicating the above conclusion. To confirm that the release of cytochrome *c* (observed in Fig. 2a, b) is a direct consequence of the BimBH3-induced BAX activation, we undertook to perform FRAP measurements on giant unilamellar vesicle (GUV) samples mimicking the lipid compositions of MOM^18,26,27^. As GUV provides a lipid environment devoid of the mitochondrial proteins, it allows a clear demonstration for the cause of the cytochrome *c* release. Briefly, GUVs were incubated with the proteins of interest over a period of time (Fig. 2c), followed by photobleaching within individual GUVs. A series of fluorescent images were collected over time after the photobleaching and then analyzed to yield the diffusion coefficients of Fluor molecules (including FITC-labeled 70-kDa, and Alexa-647-labeled 10-kDa probes) using the theoretical approach detailed in the “Methods” section. As Fluor molecules are homogeneously dispersed in the solution (outside and inside of GUVs) before the incubation with BAX and activators, it is reasonable to expect that the larger the induced pore area on the surface of a GUV, the greater the diffusion coefficient. Thus, the diffusion coefficient is directly related to the pore-forming activity of activated BAX. As the pore-forming activity is proportional to the concentration of BAX^18^, we fixed the total amount of added BAX in all of the measurements below (see the “Methods” section).

Our control experiments (lanes 1–3, Fig. 2c) showed that neither BAX nor EEE, in the absence of pro-apoptotic BimBH3, is able to induce large membrane pores on GUV. When BAX proteins were added, they were activated by BimBH3 to induce membrane pores sufficiently large for 10- and 70-kDa F-dextran molecules to pass through. However, the pore-forming ability of BAX is clearly inhibited by the presence of EEE, suggesting that BimBH3 is bound with EEE with a high affinity and thus prevented from inducing BAX activation. The ability of EEE to sequester BimBH3 is similar to that of EEE-PB, suggesting little effect of the PB-labeling to the activity of EEE. In the presence of ABT-199, BimBH3 is released from EEE to induce BAX activation and membrane pores (lane 7). These FRAP results are in a good agreement with the above cytochrome *c* (~12 kDa) release assays and, moreover, provide quantitative data for the pore-forming activity of BAX. Importantly, we demonstrated that in a MOM-like environment devoid of mitochondrial membrane proteins, the observed cytochrome *c* release is a direct consequence of the BimBH3-induced BAX activation.

### DEER reveals changes in structural flexibility of BCL-2

**Fig. 3:**
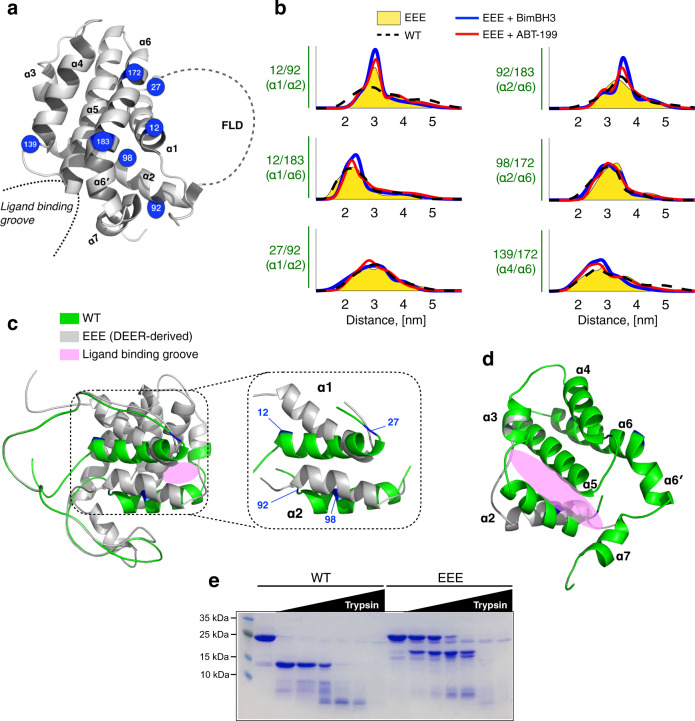
Structural differences between WT and EEE. **a** An illustration of mutation sites prepared for spin-labeling ESR studies. **b** Distance distributions of various double-spin-labeled BCL-2 (WT or EEE) mutants are determined from the experimental DEER data. EEE:BimBH3 and EEE:ABT-199 are 1:4 and 1:2, respectively. **c** Cartoon illustrations of the WT and DEER-derived EEE models. As highlighted, the displacements of α1 and α2 account for the major differences. **d** An illustration highlighting the positions of α2–α7 and the binding groove. Structures of WT and EEE are colored as in c. Helices α4–α7 change little between the WT and EEE. The displacement of α2 is critical to the change in the local environment of the binding groove. **e** Limited proteolysis of BCL-2 (20 μM) analyzed on SDS–PAGE. Trypsin concentrations were 0.04, 0.11, 0.34, 1.03, 3.11, 9.44 μM. Incubation time was 1 h at room temperature. It confirms that tertiary conformations of WT versus EEE are indeed different.

To uncover structural details of how the ligand-binding activity of BCL-2 is affected by FLD, we prepared various double-spin-labeled BCL-2 mutants (Fig. 3a, b) and performed DEER on the samples (see Supplementary Fig. 4a for raw experimental DEER data). The inter-spin distance distributions were obtained from the DEER data using the Tikhonov regularization (see the “Methods” section)^28–31^. The distance distributions of the WT mutants (black dashed lines in Fig. 3b) generally display a broad range of distances, indicating that the WT structure is characterized by a large degree of flexibility, in line with the result of a MD study on the structural dynamics of BCL-2-ΔTM^32^. This finding about the high flexibility provides an explanation of why it has been so difficult to determine the structure of FL-BCL-2 (or BCL-2-ΔTM) using other experimental tools.

The most probable distances of the distributions for each mutant (Fig. 3b) appear to change little among the studied states (i.e., WT and EEE with or without ligands). However, a comparison of the results between WT and EEE reveals some noticeable differences (cf. 12/92, 27/92, 92/183, and 98/172 in Fig. 3b) in the width of distance distributions. Among the largest differences between WT and EEE is the result of 12/92; the full-width-at-half-maximum values for the WT and EEE are 1.81 and 0.46 nm, respectively. Distances of EEE 12/92 appear to be more homogeneously distributed as compared to the broad distance distribution of WT 12/92, suggesting that prior to the ligand binding, the structural flexibility is already affected and reduced by the phosphomimetic mutations on the FLD. After adding ligands (BimBH3 or ABT-199) to the EEE solution, we observed (cf. 12/92, 12/183, 92/183) a further increase of the homogeneity (i.e., a decrease in the width) in the distance distributions. It indicates that the amplitude of dynamics (as reflected by the distribution width) in EEE is less than in WT, and it decreases further when either of the two ligands binds to EEE.

A structural model based on the DEER results of the ligand-bound state of EEE was thus derived (Fig. 3c), showing that the major differences between the WT and the ligand-bound EEE structures are due to the displacements of α1 and α2. It is primarily the displacement of α2 (as displayed in Fig. 3d) that gives rise to a change in the binding groove, hence affecting the affinity for ligands. Little change was observed for helices α4–α7 between the WT and EEE structures. Simulated inter-spin distance distributions (gray histograms in Supplementary Fig. 4b), which were generated based on the DEER-derived structure using the MtsslWizard program^33^, are reasonably consistent with the DEER distance distributions.

In addition, we carried out limited proteolysis by trypsin to probe the tertiary conformational difference between WT and EEE. It shows (Fig. 3e) that WT was susceptible to proteolysis and rapidly degraded to fragments, whereas EEE was relatively resistant although at high trypsin concentrations, several proteolytic fragments, whose molecular weights are distinctly different from those of degraded WT, appeared. The higher susceptibility of WT to proteolysis suggests a greater disorder and dynamics in the ternary structure. These results provide strong support for the DEER results that WT and EEE are different in structural conformation and dynamics.

### MD simulations show a decrease in the structural flexibility of EEE

**Fig. 4:**
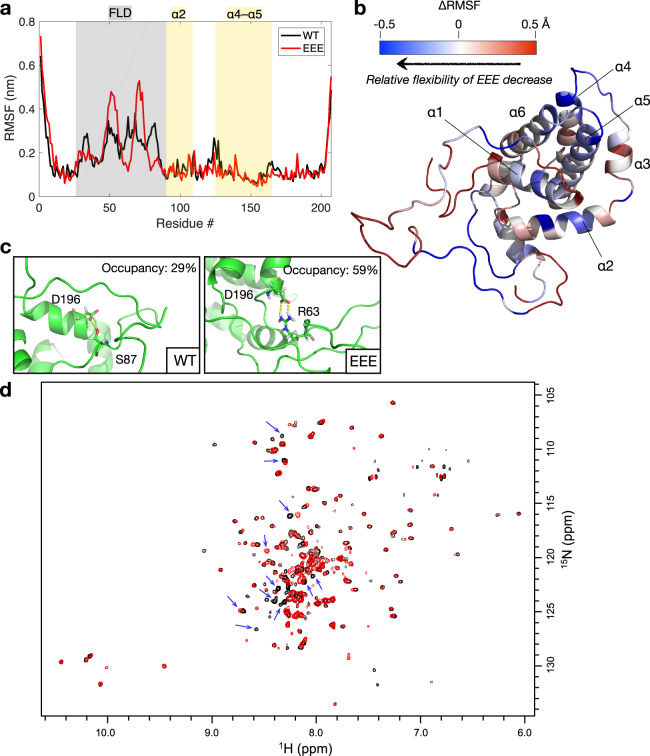
MD simulations and NMR of BCL-2 structure dynamics. **a** Per-residue RMSF result of the MD simulations (see also Supplementary Fig. 5). Segments corresponding to the FLD, α2, and α4–α5 are highlighted. **b** Cartoon illustration of the ΔRMSF results, calculated by RMSF_EEE_−RMSF_WT_. ΔRMSF < 0 (in blue) indicates a decrease in the structural flexibility as compared to WT. **c** Two hydrogen bonds can be observed in the MD trajectories. **d** The overlaid 2D ^1^H–^15^N TROSY-HSQC spectra acquired from EEE (black) and WT (red) at 298 K. Blue arrows indicate the differences caused by Gly residues and several residues in the un-structured region.

We performed MD simulations to explore the structural dynamics of WT and EEE. Geometric parameters, root mean square deviation (RMSD) and radius of gyration (*R*_G_), were used to evaluate the stability of systems and the simulation convergence (Supplementary Fig. 5). The per-residue RMS fluctuation (RMSF) analysis, over the equilibrated period (>500 ns), (Fig. 4a) and the corresponding cartoon illustration (Fig. 4b) reveal the differences in the dynamical fluctuations (structural flexibility) between the WT and EEE structures. The relative RMSF (ΔRMSF) results indicated that the three phosphomimetic mutations not only disturb the flexibility of the FLD (gray shaded) but also cause some reduction in the dynamics of structured regions (particularly in the helices of α2, α4, and α5, as shaded in yellow in Fig. 4a). That MD result is in line with the DEER result.

In analyzing the occupancy of hydrogen bonds from the MD trajectory, we observed a maximum hydrogen bond occupancy of 29% between D196 and S87 in the WT simulation. In the EEE simulation, we observed a salt bridge between D196 and R63 with a hydrogen bond occupancy of 59% (Fig. 4c). The newly formed salt-bridge may play a role in reducing the flexibility of the EEE structure. In the context of phosphorylation modifications (or phosphomimetic mutations), which add negative charges to the surface of protein, the modifications on the FLD would cause favorable electrostatic interactions with positive charges elsewhere on the protein and thus reduce the dynamics of the structured regions. Our MD results support an important role of the modification of the FLD in determining the structural flexibility of BCL-2.

Together, the DEER and MD results show that the structural flexibility of BCL-2 is decreased in the phosphomimetic mutant EEE. To further support the finding, we performed NMR to record TROSY-HSQC spectra (Fig. 4d) of the ^15^N-labeled EEE alone, WT alone, and then TROSY-HSQC spectrum of the EEE in the presence of 2-fold ABT-199 (Supplementary Fig. 6). Without sequential assignment of the resonances, we cannot characterize individual residues. However, there are significant and apparent differences between the spectra of WT and EEE (Fig. 4d). Some Gly residues (with ^1^H_N_ at 8.0–8.5 ppm and ^15^N at 108–113 ppm) and several residues in the un-structured region (with ^1^H_N_ at 8.0–8.5 ppm and ^15^N at 115–130 ppm) exhibit perturbed resonances between the two proteins (indicated by blue arrows in Fig. 4d). As there are two Gly residues (79 and 83; Supplementary Fig. 1) located between the EEE mutations (69, 70, and 87), they may account for part of the spectral changes observed. Since EEE mutations are located in the FLD, they also affect the NMR signals from residues in the un-structured region. This observation is further strengthened by the existence of two sets of resonances for some residues in the WT spectrum. The EEE spectrum has better peak shape and higher peak intensity as compared to the WT spectrum, supporting our DEER result that EEE has a more constrained structure in solution.

## Conclusion

**Fig. 5:**
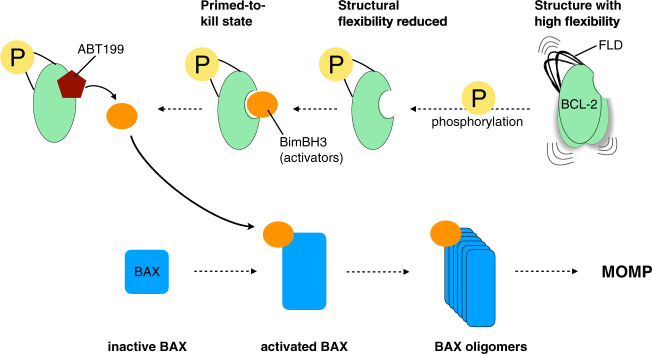
Model proposed for the allosteric regulation of BCL-2. BCL-2 WT is characterized by a large degree of flexibility in structure. Our results suggest that the modification on the FLD improves the structural homogeneity and reduces the flexibility. The reduced flexibility in the FLD, which is distal to the core structure, mediates allosterically the ligand-binding groove, increasing the affinity for BimBH3 activators. The bound activators can be displaced by ABT-199, causing the release of BimBH3 and subsequently trigging BAX-induced MOMP.

Our findings, summarized in Fig. 5, support an allosteric model for the regulation of BCL-2 anti-apoptotic activity by changes in structural flexibility of the 65-residue-long unstructured FLD. We reveal that BCL-2 WT structure is characterized by large-amplitude dynamics, as evidenced by both the DEER and MD results. After phosphorylation (or phosphomimetic) modification on the FLD, the amplitude of structural dynamics is decreased. This decrease is critical to the change in the ligand-binding groove environment (surrounded by α2–α5), consequently promoting the affinity of BCL-2 for activators (e.g., BimBH3). The BimBH3-bound state of BCL-2 EEE is considered as a “primed-to-kill” state as it is inactive in causing MOMP, whereas it can be efficiently triggered by ABT-199 to release the bound pro-apoptotic activators and cause lethal MOMP. We reveal that changing phosphorylation state on the FLD leads to a coordinated response predominately in the structure flexibility, which is directly linked to the anti-apoptotic function of BCL-2. Our work offers new insights into how a change in structural flexibility of a highly flexible loop can modulate protein conformation and consequently determine whether protein–ligand interactions will occur.

## Methods

### Recombinant protein preparation

#### BCL-2 expression and purification

Human BCL-2∆TM (lacking TM domain) (GenBank BC027258; residues 1–217) was cloned into NdeI/XhoI site of pET22b(+) vector (New England Biolabs, Inc.), generating the BCL-2-∆TM protein with a C-terminal His6-tag. Plasmids encoding BCL-2 mutants were generated using a QuikChange mutagenesis kit at the indicated sites and verified by DNA sequencing. BCL-2 were expressed and purified by an affinity Ni-column, as described^34^. Briefly, the plasmid was transformed into the *E. coli* BL21(DE3) expression strain (Novagen) by heat shock. Bacterial culture was grown at 37 °C in Luria–Bertani (LB) medium containing 0.1 g/mL ampicillin until reached to OD600 above 1.0. Protein expression was induced by addition of 0.3 mM isopropyl 1-thio-β-d-galactopyranoside (IPTG) at 30 °C for 4–6 h. The cells were harvested by centrifugation, resuspended in ice-cold lysis buffer PEB (50 mM Tris (pH 7.4), 0.2 M NaCl, 5 mM β-ME, 5 mM imidazole, 10% (v/v) glycerol, and protease inhibitor tablet (cOmplete)), and stirred for 20 min at 4 °C. Lysozyme (0.2 mg/mL) and a pinch of DNaseI were added to the cell mixture, and the solution was stirred for an additional 20 min. The resuspended pellet was sonicated by pulse sonication at 70% output on ice for 10 min, followed by centrifugation at 30,000 × *g* for 15 min. The soluble fraction was filtrated through a 0.22 mm filter and loaded onto an affinity Ni-column using HisTrap HP (GE Healthcare) pre-equilibrated with PEB, and unbound proteins were removed with 10 column volumes (CV) of PEB containing 40 mM imidazole. Proteins were eluted with 10 CV of PEB containing 200 mM imidazole, desalted by PD-10, and further purified by a size-exclusion chromatography (SEC) using a HiLoad 16/60 Superdex 75 column (GE Healthcare) pre-equilibrated in PB buffer (20 mM sodium phosphate, pH 7.4, 100 mM NaCl). Purified proteins were confirmed by sodium dodecyl sulfate–polyacrylamide gel electrophoresis (SDS–PAGE) with Coomassie blue staining. Protein concentration was determined using absorption spectroscopy at 280 nm. BCL-2 WT has a native cysteine 158, which was used for conjugating with fluorescence probe. Cysteine-free construct BCL-2 (i.e., WT C158A) was used to prepare single-cysteine or double-cysteine variants of BCL-2 for spin-labeling study. Previous studies concerning the mono- and multisite phosphomimetic mutations of BCL-2 can be found elsewhere^5,9–11^.

#### BAX expression and purification

Full-length BAX and variants were prepared as previously described^18,22,35^. Mouse BAX was cloned into pTYB1 vector (New England Biolabs, Inc.), resulting pTYB1-BAX construct. The pTYB1-BAX construct encoding a fusion protein of BAX with chitin-binding peptide was separated by a self-cleavable intein tag to obtain a full-length BAX. Wild-type BAX has two native cysteine residues at 62 and 126. Cysteine-free construct (C62S/C126S) was used to prepare single-cysteine BAX variants for conjugating fluorescence probes (e.g., BAX136C in Fig. 1d). Point mutations of recombinant BAX were generated using a QuikChange mutagenesis kit. Proteins were expressed in *E. coli* (ER2566) strain (New England Biolabs, Inc.) and purified without detergent. Bacterial cultures were grown at 37 °C in LB medium containing 0.1 g/L ampicillin to reach an OD600 of 1.0. BAX expression was induced by addition of 0.3 mM IPTG at 30 °C for 6−8 h. The cells were harvested by centrifugation and resuspended in ice-cold lysis buffer (20 mM Tris–HCl, pH 7.0, 500 mM NaCl and 1 mM PMSF). The resuspended pellet was sonicated on ice for 30 min, followed by centrifugation at 12,800 × *g* for 50 min. The soluble fraction was loaded onto chitin affinity resin column at a flow rate of 0.5 mL/min, and unbonded proteins were washed with lysis buffer. The resins were incubated in a pre-equilibrated buffer (20 mM sodium phosphate, pH 8.0, 100 mM NaCl, 60 mM dithiothreitol (DTT)) for 48 h. Proteins were eluted from column and further purified by a SEC using a HiLoad 16/60 Superdex 75 column (GE Healthcare) pre-equilibrated in PB buffer (20 mM sodium phosphate, pH 7.4, 100 mM NaCl). Purified proteins were confirmed by SDS–PAGE with Coomassie blue staining. Protein concentration was determined using absorption spectroscopy at 280 nm. A typical yield (>95% purity) of 0.15 mg/L could reproducibly be obtained for BAX.

To prepare cleaved Bid (cBid) and T4 lysozyme (T4L), we followed the protocols that we previously published^36,37^.

### Preparation of pro-apoptotic BimBH3 peptide

Synthesis of BimBH3 peptides was performed with solid‐phase methods using Fmoc‐protected amino acids, HBTU‐mediated coupling, rink amide AM resin (Creosalus), and standard reaction cycles on a PS3 (Proteins Technologies) synthesizer. For the coupling of natural Fmoc-protected amino acids (Creosalus), 4 equivalent of amino acid and a 1:1 molar ratio of coupling reagents O-benzotriazol-1-yl-N,N,N′,N′-tetramethyluronium hexafluorophosphate (HBTU)/either 1-hydroxybenzotriazole (HOBt) (Creosalus) were used. A 4 mL cocktail solution containing 94.0% trifluoroacetic acid (TFA), 1.0% triisopropylsilane (TIS), 2.5% ethanedithiol (EDT) and 2.5% H_2_O (v/v) was used to cleave the peptides from resin and to remove the side chain protecting groups. The crude peptides were precipitated by adding precooled methyl tert-butyl ether and the precipitates were collected by centrifugation. Peptides were dissolved in 50% (v/v) acetonitrile (ACN), followed by the purification using reverse phase HPLC with semi-preparative C18 reversed phase HPLC columns on 60 min linear gradient from 0% to 50% buffer B (A: H_2_O, 0.1% TFA; B: 99.9% ACN, 0.1% TFA) at a constant flow rate of 3.0 mL/min. Identity of the peptide was confirmed by MALDI-TOF mass spectrometry. Sequence of BimBH3 is as follows: H2N-DMRPEIWIAQELRRIGDEFNAYYAR-amide. We added a cysteine at the C‐terminus of BimBH3 for the purpose of spin or fluorescence labeling. The use of Rink amide resin generated an amidated C-terminus upon cleavage.

### Mitochondria isolation and cytochrome c release assays

A Dounce homogenizer was used with 20 strokes to homogenize mouse liver tissue (0.2 mg). The mitochondria fraction was separated using centrifugation according to the Mitochondria Isolation Kit (Thermo Scientific). Mitochondria Assay Buffer A (MAB A) (200 mM mannitol, 68 mM sucrose, 10 mM HEPES, 110 mM KCl, 1 mM EDTA, 1 mM EDTA, 0.1% BSA, protease inhibitor) was used to wash the mitochondria fraction, followed by centrifugation at 12,000 × *g*. The pellet was collected and resuspended using enough MAB B (i.e., MBA A without 0.1% BSA) and then quantified by bradford assay (Bio-Rad) to yield a final protein content of the mitochondrial fraction ~3 mg/mL. The final mitochondria fraction was placed on ice for further downstream processing or stored at −80 °C with additional 300 mM trehalose for future use^38^. For the cytochrome *c* release assay, BimBH3 (2 μM) was incubated with BCL-2 WT, or mutants first for 0.5 h at room temperatures in PB buffer, followed by, when necessary, addition of ABT-199 at varying concentrations (i.e. 4–30 μM) for another 0.5 h at room temperatures. The mixture was then incubated with BAX (0.1 μM) and 10 μL of the mitochondrial fraction (100 μg of protein) for 0.5 h at 37 °C. Final volume was adjusted to 30 μL. Following centrifugation at 12,000×*g* for 10 min, the supernatant was subjected to subsequent immunoblotting (IB). Quantification of IB was done using ImageJ software (version 1.49, NIH). The fraction of cytochrome *c* released (%) is calculated by (*I*–*I*_0_)/(*I*_max_–*I*_0_)×100%, where *I*_max_ and *I*_0_ are the analyzed intensities of cytochrome *c* with and without activated BAX, respectively.

### Immunoblotting

Samples were resolved by SDS–PAGE (15%) and electroblotted onto a 0.45 μm PVDF membrane (GE Healthcare). Primary antibodies used in this study included rabbit anti-BAX N20 (Santa Cruz Biotechnology), mouse anti-BCL-2(C2) (Santa Cruz Biotechnology) and mouse anti-cytochrome *c* antibodies (clone 7H8.2C12, Millipore). Detection was achieved using sheep anti-mouse IgG HRP (GE Healthcare) and donkey anti-rabbit IgG HRP (GE Healthcare) secondary antibodies. Proteins were visualized by 4CN Plus Chromogenic Substrate (PerkinElmer).

### FRET measurements and analysis

Proteins and BimBH3 peptide were site-specifically labeled at engineered cysteines using maleimide chemistry. PB dye (Invitrogen) and AlexaFluor 488 (Invitrogen) were used as FRET donor and acceptor, respectively. Purified proteins or BimBH3 peptide were labeled with a 2-fold molar excess of probe in PB buffer for overnight at 4 °C. For proteins, excess probe was removed by PD-10 with PB buffer. Unreacted and reacted peptide were separated by reverse phase HPLC using semi-preparative C18 reversed phase HPLC columns. The identity of the peptide was confirmed by MALDI-TOF mass spectrometry. Protein and peptide concentrations were determined using absorption spectroscopy at 280 nm with adjusting by a correction factor (CF) which has been listed in Thermo Scientific TECH TIP #31. In a black polystyrene NCNU 96-well plate (Thermo Scientific), donor-conjugated proteins (1 µM) were incubated with acceptor-conjugated proteins (2 µM) or conjugated BimBH3 peptide (2 µM) in PB buffer for 1.5 h at room temperature. To examine whether ABT-199 impedes the interaction between BCL2 and BimBH3 (Supplementary Fig. 2), donor-conjugated BCL-2 (WT-PB or EEE-PB) was incubated with the indicated concentration of ABT-199 for 1.5 h at room temperature before adding acceptor-conjugated BimBH3 peptide (BimBH3-AL488). To verify the FRET signals of EEE-PB and BimBH3-AL488, EEE-PB was incubated with indicated concentration of unconjugated BimBH3 for 1.5 h at room temperature before adding BimBH3-AL488. Fluorescence spectra (440–640 nm) were recorded using a Synergy H1 multi-mode microplate reader (BioTek) at indicated time with excitation at 410 nm. FRET efficiency is calculated by *E* = 1–(*I*/*I*_0_), where *I* and *I*_0_ are the measured donor fluorescence intensities at 455 nm in the presence and absence of acceptor, respectively.

### Preparation of GUVs

All lipids used were purchased from Avanti Polar Lipids. The lipid mixture mimicking the MOM composition was prepared with 4% bovine heart cardiolipin (CL), 10% l-α-phos-phatidylinositol (PI), 10% 1-palmitoyl-2-oleoyl-sn-glycero-3-phospho-l-serine (POPS), 28% 1-pal-mitoyl-2-oleoyl-sn-glycero-3-phosphoethanolamine (POPE), and 48% 1-palmitoyl-2-oleoyl-sn-glycero-3-phosphocholine (POPC) in mole percentage. Texas Red 1,2-dihexadecanoyl-sn-glycero-3-phosphoethanolamine (TR-DPPE) (Invitrogen) of 0.05% was added to the lipid mixture for virtualization of GUV^18,26,27^. GUVs were prepared with the gel-assisted swelling on a polyvinyl alcohol (PVA) gel^39^. A 5% (w/w) solution of PVA (MW 145,000) was prepared by stirring PVA in ddH_2_O while heating at 90 °C. PVA solution was spread on a microscope coverslip (18 × 18 mm, Matsunami, Japan) and then dried at 50 °C. Lipid mixture (1 mg/mL) dissolved in chloroform were spread on the dry PVA film and placed under vacuum for 30 min to evaporate the solvent. Lipids were rehydrated for 1 h in PB buffer. The resulting GUVs were transferred to an Eppendorf tube using blunt tips. This study verified that these GUVs prepared with the above protocol are stable in the buffers used and they remain intact for a long time (>24 h)^18^.

### FRAP measurements and analysis

FRAP measurements were performed as described previously^18^. BAX (1 μM) was incubated with BimBH3 peptide (2 μM) and 100 μL GUVs suspensions in NuncTMLabTekTM chambered coverglass (Thermo Scientific) for 1 h as a positive control. To evaluate the BCL-2 function, BCL-2 (e.g., EEE or EEE-PB) (1 μM) and BimBH3 (2 μM) were incubated with GUV suspensions for 1 h, followed by the addition of BAX (1 μM) and incubation for another 1 h. To assess the effect of ABT-199 to BCL-2, ABT-199 (30 μM) was incubated with the mixture (BCL-2, BimBH3, and GUVs suspensions) for 1 h before the addition of BAX. All reactions were at room temperature and the final volume was 200 μL. FRAP measurements were performed with a confocal scanning laser microscopes (CSLM) (LSM700 confocal microscopy, Carl Zeiss). To capture the image, ×40 objective was selected, nominal speed 10 was used, and the pinhole was open completely to detect as much fluorescence light as possible with a low power illumination beam to minimize bleaching when recording the fluorescence recovery. After a disk of a particular diameter has been drawn in the bleaching software, time-series images were recorded with the CSLM to obtain a stack of images at a time interval of 4 s. Totally, a stack of 50 images was collected for every GUV. The experimental recovery curves were extracted from the image stack and analyzed using the Matlab program (R2018a, MathWorks), as previously demonstrated^18^, to calculate the diffusion coefficients of the added fluorophore-conjugated dextran (F-dextran) molecules. The pore-forming activity was defined by *D*/*D*_0_, where *D*_0_ is the diffusion coefficient measured after adding 0.5% Triton X-100 into the buffer containing GUVs. *D*_0_ values are 26.5 μm^2^/s (70-kDa F-dextran) and 51.2 μm^2^/s (10-kDa F-dextran). *D*/*D*_0_ would approach to 1 when GUVs are largely disrupted into pieces. In general, the larger the induced pore area, the greater the FRAP rate is obtained, which indicates a greater pore-forming activity. As *D*/*D*_0_ also depends on the amount of the added BAX, the concentration of BAX (1 μM) was fixed in all FRAP measurements.

### Limited proteolysis

BCL-2 conformational change was assessed by limited proteolysis using trypsin (MERCK). In brief, 5 µg BCL-2 (WT or EEE) was digested at enzyme/substrate ratios ranging from 1:2 to 1:461 for 1 h at room temperature. Proteolysis was quenched by adding 1 mM PMSF, and then samples were confirmed by SDS–PAGE with Coomassie blue staining.

### DEER measurements

Purified BCL-2 proteins containing 5% (v/v) glycerol were labeled with a 10-fold molar excess of MTSSL per cysteine residue in the dark for overnight at 4 °C. Excess MTSSL was removed by PD-10 with D_2_O buffer (20 mM sodium phosphate, 100 mM NaCl, 2.5% glycerol, pH 7.4). 4-fold BimBH3 peptide (or 2-fold ABT-199) was mixed with BCL-2 (0.3 mM) and incubated at room temperatures for 1 h. Approximately, 40 μL solution volume, containing 30% (v/v) d8-glycerol as cryoprotectant, was added into a quartz ESR tube (3 mm) and flash-cooled in liquid nitrogen before being transferred into the ESR probe head. A Bruker ELEXSYS E580-400 pulsed spectrometer, with a split-ring resonator (EN4118X-MS3) and a helium gas flow system (4118CF and 4112HV), was used. ESR probe head (ER4118X-MS3) was precooled to 80 K using a helium flow system prior to the transfer of the ESR sample tube into the cavity. DEER experiments were carried out with the typical four-pulse constant-time DEER sequence^18,21^. Detection pulses were 32 ns (*π*) and 16 ns (*π*/2). The pump frequency was set to approximately 70 MHz lower than the detection pulse frequency. The pulse amplitudes were chosen to optimize the refocused echo. The π/2-pulse was employed with +x/−x phase cycle to eliminate receiver offsets. The duration of pumping pulse was about 32 ns, and its frequency was coupled into the microwave bridge by a commercially available setup (E580-400U) from Bruker. All pulses were amplified via a pulsed traveling wave tube (TWT) amplifier (E580-1030). The field was adjusted such that the pump pulse is applied to the maximum in the nitroxide-based MTSSL spectrum. Accumulation time for each set of data was 10 h at 80 K. Inter-spin distance distributions were obtained from the experimental DEER time-domain data using the Tikhonov regularization method based on the L-curve criterion, followed by a data refinement process using the maximum entropy method^21,28,29^.

### Circular dichroism (CD) spectroscopy

CD measurements were conducted on an AVIV Model 410 CD spectrometer. Far-UV CD spectra were recorded using a 1 mm path length quartz cuvette. Proteins were dissolved in PB buffer with a final concentration of 5–10 μM. Spectra were recorded from 260 to 195 nm (Far-UV) with a slit width of 1 nm, and signals were averaged for 10 s at each wavelength. The ellipticities of temperature-dependent CD signals were recorded in the range from 25 to 95 °C with an average heating rate of 0.33 °C/min and an interval of 5 °C. All spectra shown were background-corrected by subtracting corresponding blanks.

### MD simulation and analysis of MD trajectories

The atomistic structure of BCL-2 WT obtained from protein model database (code PMDB PM0077081) was used in the simulation^40^. Both WT and EEE mutants were modeled using PSFGEN utility of VMD^41^. The system was soaked in AMBER-ff99SB-disp water boxes and neutralizing ions (Na+ and Cl−) at an experimental concentration (100 mM) using the Gromacs 2020.1 program^42,43^. The simulation was performed using the Gromacs program with the AMBER-ff99SB-disp force field. Periodic boundary conditions^44^, particle mesh Ewald method^45^, LINCS algorithm for fixing all bonds linking hydrogen atoms^46^, a non-bonded cutoff of 1.2 nm, and a 2 fs time step were used. The system was heated with v-rescale temperature coupling, using an NPT ensemble, to the simulation temperature 300 K. The solvated proteins were performed steepest descent minimization with 2000 steps, then run 1500 ns for unbiased molecular dynamics simulation after 300 ps for constant pressure equilibration at 300 K. Coordinates, energies, and pressures were saved for analysis every 2 ps. The homemade VMD Tcl code, and VMD’s plugins were used to analyze R_G_, RMSD, and RMSF about the mean position of Cα atoms.

### NMR measurements

For NMR measurements, the ^15^N-labeled recombinant EEE proteins were expressed in *E. coli* supplemented with ^15^N-ammonium chloride. Bacteria were grown in LB with 100 μg/mL ampicillin at 37 °C overnight^47^. Once the cell density reaches OD_600_ of 3−5 in LB, cell culture was centrifuged at 5000 × *g* for 10 min and resuspended in the same volume of M9+ medium with 0.01% thiamine, ampicillin (0.1 g/L), and ^15^NH_4_Cl (1 g/L)^48^. Protein expression was induced by the addition of 1 mM IPTG after incubation at 37 °C for 2 h. After 20 h at 25 °C induction period, the cells were harvested by centrifugation. The purification procedures were the same as described above. For WT, the second purified column was 5 mL HiTrap Q HP anion exchange chromatography column (Q-column) (GE Healthcare) instead of SEC. Sample was loaded onto Q-column equilibrated in 20 mM Tris (pH 7.6) and eluted by an NaCl step gradient with an elution buffer (20 mM Tris, pH 7.6, 1 M NaCl). The flow rate for binding and elution was 5 mL/min. Then, sample buffer was exchanged into PB buffer using PD-10 desalting column. Purification of WT was confirmed by SEC and SDS–PAGE with Coomassie blue staining. NMR spectra were acquired on a Bruker AVANCE III 850 spectrometer at 298 K. The ^15^N-labeled samples (0.1 mM) were prepared in NMR buffer (PB buffer with 5% glycerol, 1 mM EDTA, 5 mM DTT, 1x protease inhibitor cocktail (Roche), and 10% D_2_O (v/v)) with or without 2-fold ABT-199.

### Statistics and reproducibility

This manuscript is accompanied by a file that contains all raw data from the studies contained in this work. The statistical analyses were based on a one-way ANOVA test (^n.s^*p* > 0.05; *p ≤ 0.05; **p ≤ 0.01; ***p ≤ 0.001). The exact *p* values are indicated either directly in the figure or in the legend. The exact sample size is given in the legend of each figure. The mean ± standard deviation (SD) is displayed, unless otherwise stated. The standard deviation is not displayed if it is smaller than the corresponding symbol.

### Reporting summary

Further information on research design is available in the Nature Research Reporting Summary linked to this article.

## Supplementary information

Supplementary Information

Description of Additional Supplementary Files

Supplementary Data

Reporting Summary

## Data Availability

The authors declare that all data supporting the findings of this study are available within this article and its supplementary information files or from the corresponding author upon reasonable request. The source data for the figures are in the Supplementary Data.
