# Supplementary material for: Anti-apoptotic BCL-2 regulation by changes in dynamics of its long unstructured loop
Source: Commun Biol. 2020 Nov 12;3:668. doi: 10.1038/s42003-020-01390-6 (PMC7665024; doi:10.1038/s42003-020-01390-6)
Supplement: Supplementary file 1 — Supplementary Information [file 42003_2020_1390_MOESM1_ESM.pdf]

Supplementary Information for

Anti-apoptotic BCL-2 regulation by changes in dynamics of its long unstructured loop

Yu-Jing Lan,†[a] Pei-Shan Yeh,†[a] Te-Yu Kao,[a] Yuan-Chao Lo,[b] Shih-Che Sue,[c] Yu-Wen Chen,[d] Dennis W. Hwang,*[d] and Yun-Wei Chiang*[a]

[a]Department of Chemistry, National Tsing Hua University, Hsinchu, Taiwan

[b]Biomedical Translation Research Center, Academia Sinica, Taipei, Taiwan

[c]Institute of Bioinformatics and Structural Biology, National Tsing Hua University, Hsinchu, Taiwan

[d]Institute of Biomedical Sciences, Academia Sinica, Taipei, Taiwan

*Corresponding email: ywchiang@gapp.nthu.edu.tw; dwhwang@ibms.sinica.edu.tw

†These authors contributed equally to this work.

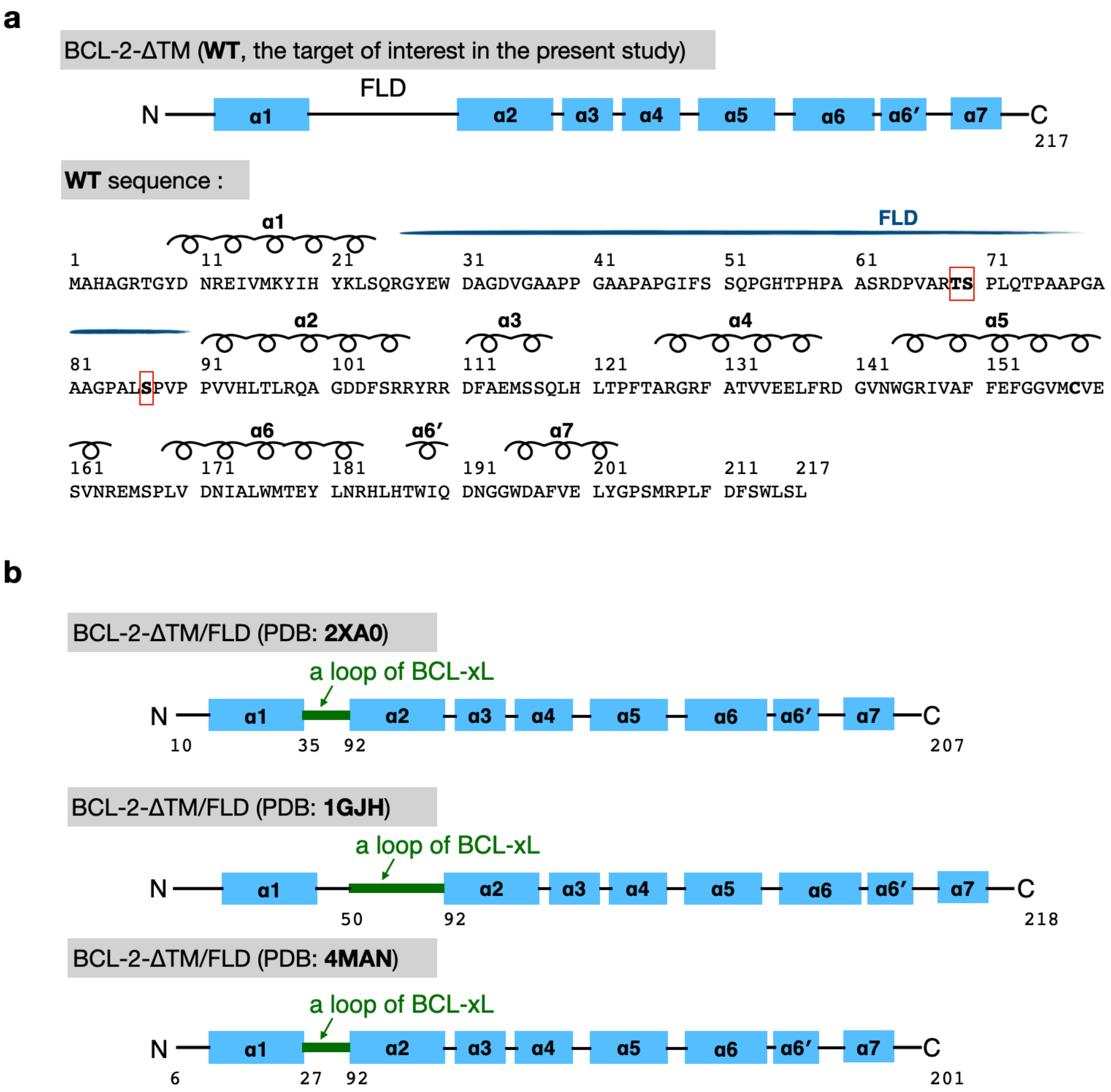

Supplementary Fig. 1: Sequences of the truncated BCL-2 proteins. a Sequence of BCL-2-ΔTM (denoted by WT in the present study). Red boxes indicate the three phosphorylation-related residues T69, S70, and S87. **b** A comparison of the sequences among various truncated forms of BCL-2 whose structures have been determined previously using NMR or X-ray methods. They include (PDB code) 2XA0, 1GJH, and 4MAN, all of which lack the intrinsic 65-residue-long FLD and the TM (α8) helix.

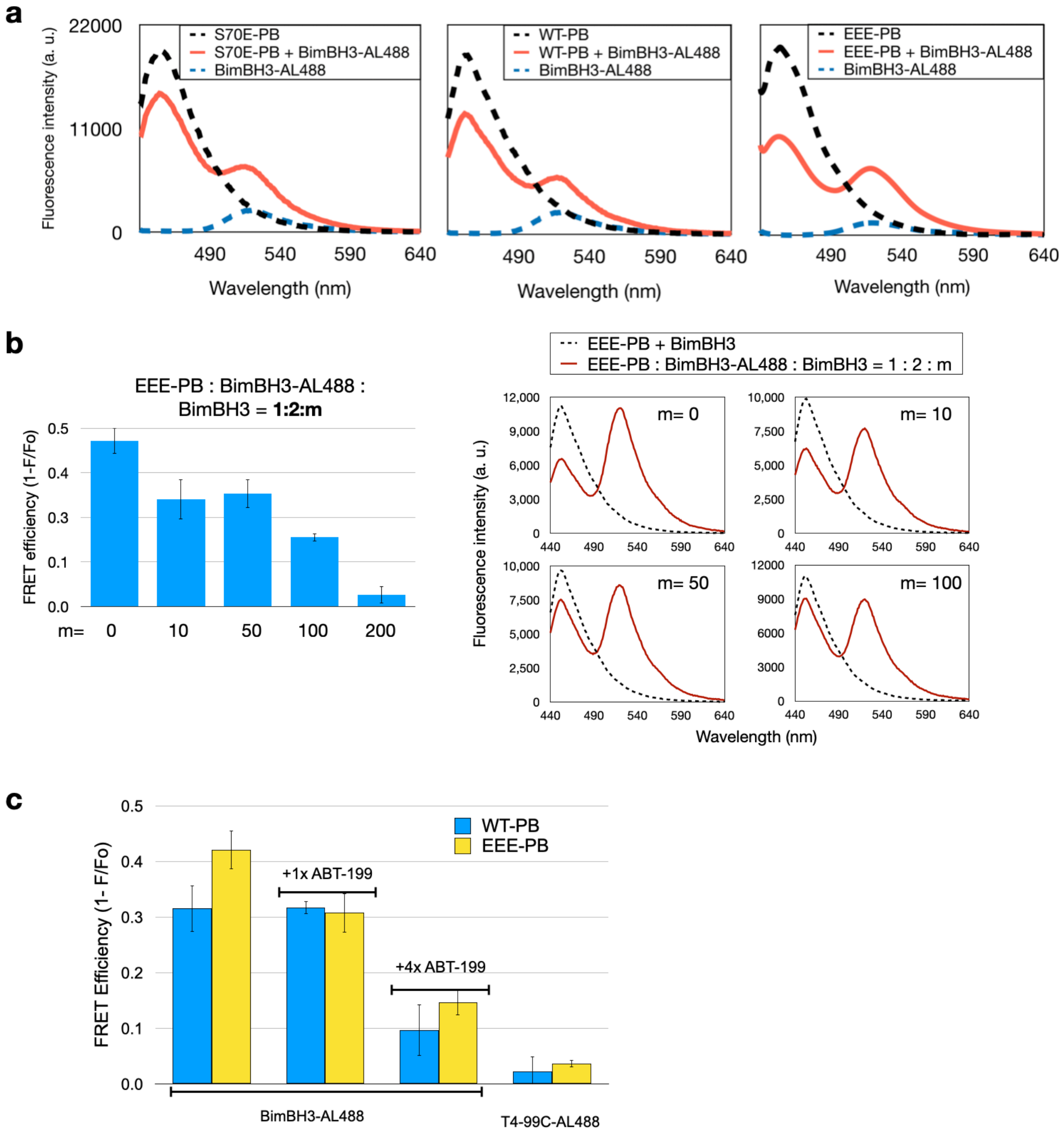

Supplementary Fig. 2: FRET results. a Some representative data of the FRET measurements. The associations between EEE-PB and BimBH3-AL488 are clearly greater than those when EEE-PB is replaced by S70E-PB or WT-PB. The analysis result is summarized in Fig. 1d. **b** A control experiment for the above FRET data. EEE-PB was incubated with a varying amount of un-labeled BimBH3 (as indicated), followed by the addition of labeled BimBH3 (i.e., BimBH3-AL488). The results confirm that the observed FRET data reflect the association of EEE with BimBH3. **c** FRET results to verify that the

effect of ABT-199. ABT-199 was added into the solution containing BCL-2 (WT- or EEE-PB):BimBH3-AL488 1:2. The FRET efficiency decreases with increasing amounts of ABT-199. It verifies that ABT-199 can displace BimBH3 from BCL-2 efficiently. Concentrations of BCL-2 (1 μM) and BimBH3 (2 μM) are fixed.

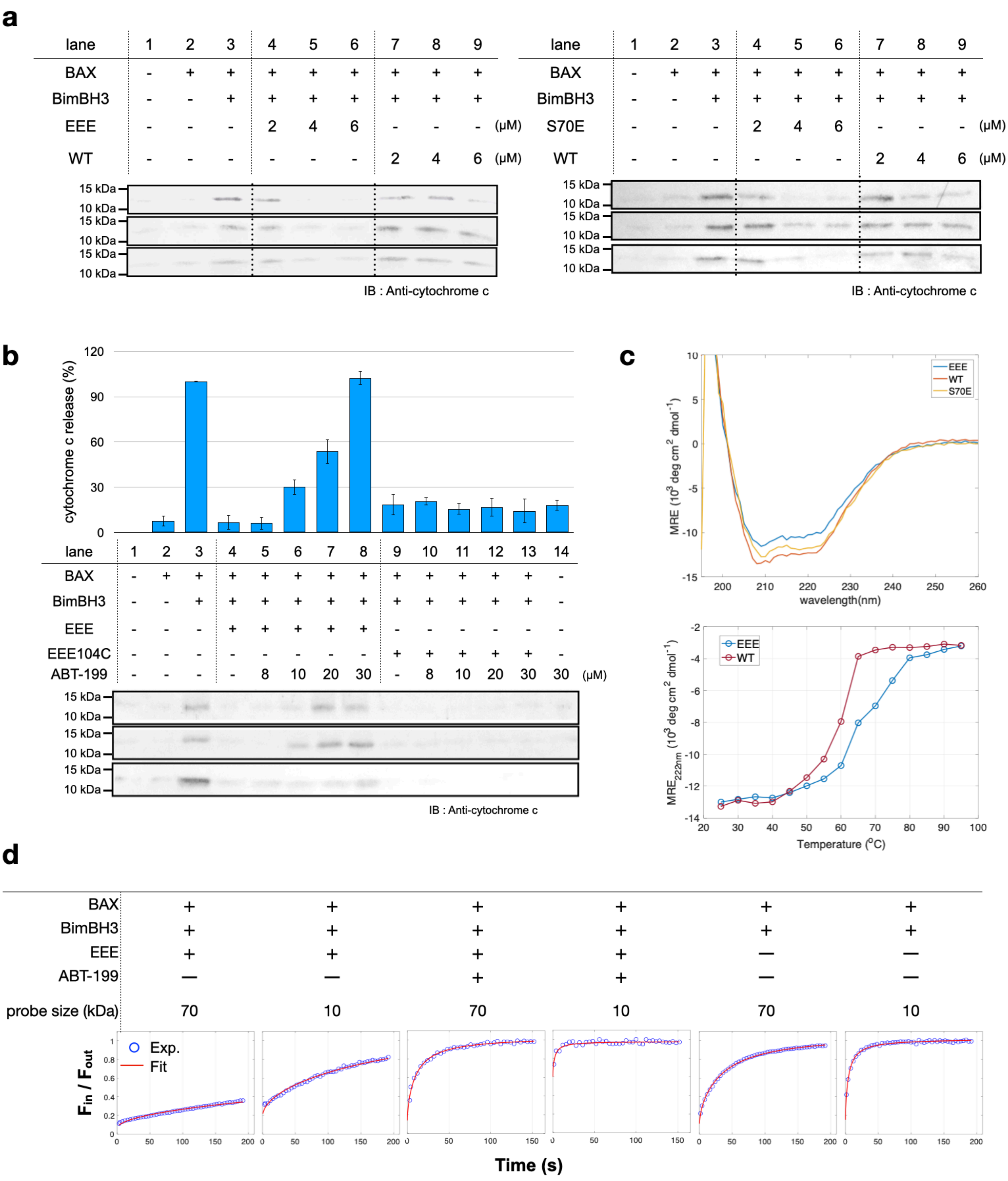

Supplementary Fig. 3: Experimental data of IB, CD, and FRAP. a Some representative results of the immunoblotting assay. The analysis of the results is summarized in Fig. 2a. **b** More supporting results of the cytochrome c release assay. They are normalized to the result of BAX:BimBH3 1:20 (lane 3). ABT-199 positively regulates the amount of the free BimBH3 (i.e., BimBH3 released by EEE) and thus promote the BAX-induced MOMP (lanes

4–8). When the ABT-199-insensitive mutant EEE104C is used (Blood_123_2014_4111), adding ABT-199 is ineffective in causing the release of cytochrome c from mitochondria (lanes 9–14). **c** CD spectra of BCL-2 WT, EEE, and S70E are similar, confirming that they retain the same secondary structure at room temperatures. Data shown are the mean residual ellipticity (MRE). Thermal denaturation (lower panel) study (25–95 °C) of WT and EEE. It shows that EEE has a slightly (ca. 5–10 °C) higher melting temperature than WT. **d** Some representative raw fluorescence recovery FRAP data (blue) and fits (red) for 10 kDa and 70 kDa (see also Fig. 2c).

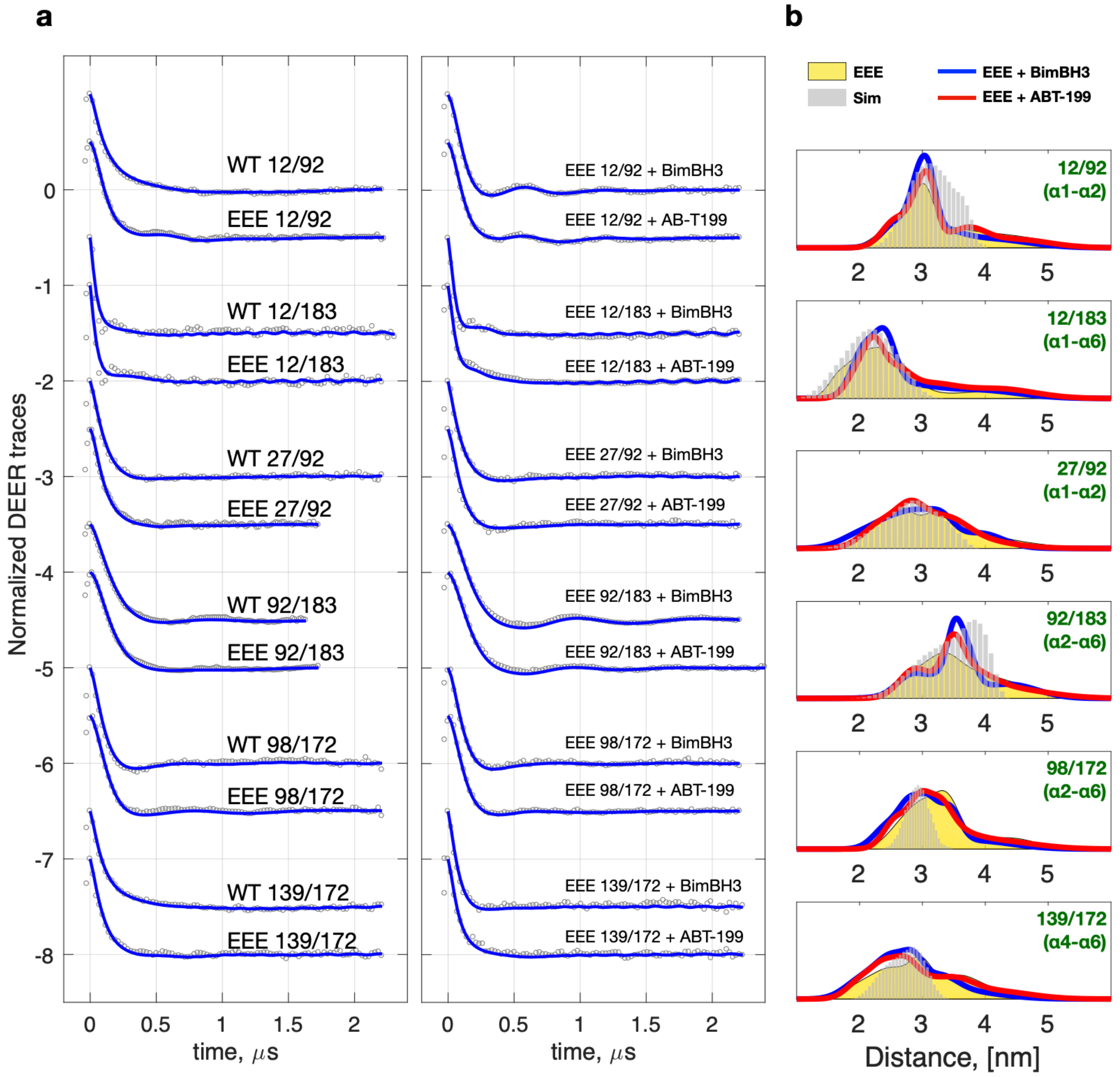

Supplementary Fig. 4: Experimental data and analysis of DEER measurements. a Normalized DEER experimental traces (gray circles) after the background removal. Simulated traces obtained using the TIKR-derived distance distributions (as shown in Fig. 3b) are plotted in blue lines. The simulated traces are in a good agreement with the experimental traces, supporting the reliability of the TIKR results. **b** A comparison of the simulated distance distributions (gray histograms), which are generated from the DEER-derived EEE model (as shown in Fig. 3c) using the MtsslWizard program, and the the TIKR-derived distance distributions of the studied EEE samples. The major peaks of the simulations

are consistent reasonably with those of the TIKR results, but the distribution widths of the former are generally less than those of the later. The discrepancy in the width is attributed to that structural flexibility is not considered in the MtsslWizard calculations.

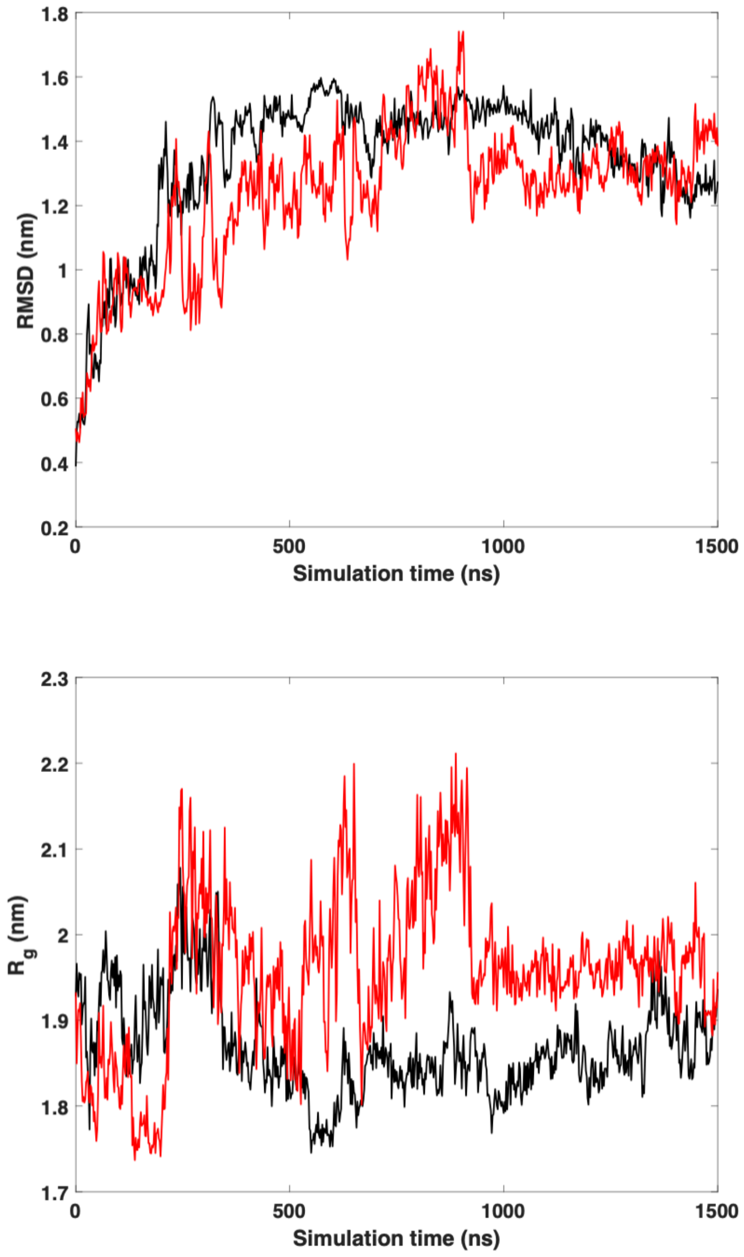

Supplementary Fig. 5: Details of the MD simulations. Simulations were carried out in explicit water for 1500 ns. The RMSD (top) and R_G (bottom) results of the WT (black line) and EEE (red line) simulations are shown above. The RMSD values of all protein atoms are calculated from the entire 1500 ns trajectory, providing a glimpse of the system stabilities of WT and EEE. It shows that the systems tend to converge after ~900 ns, with RMSD values of 1.37 ± 0.09 and 1.32 ± 0.07 nm for WT and EEE, respectively. The radius of gyration values are calculated to demonstrate a good simulation convergence. The systems converge after

~900 ns with R_G values of 1.86 ± 0.03 and 1.96 ± 0.03 nm for WT and EEE, respectively. Overall, both of the MD simulations reach convergence approximately after 900 ns and maintain the stable structures until the end of the simulations.

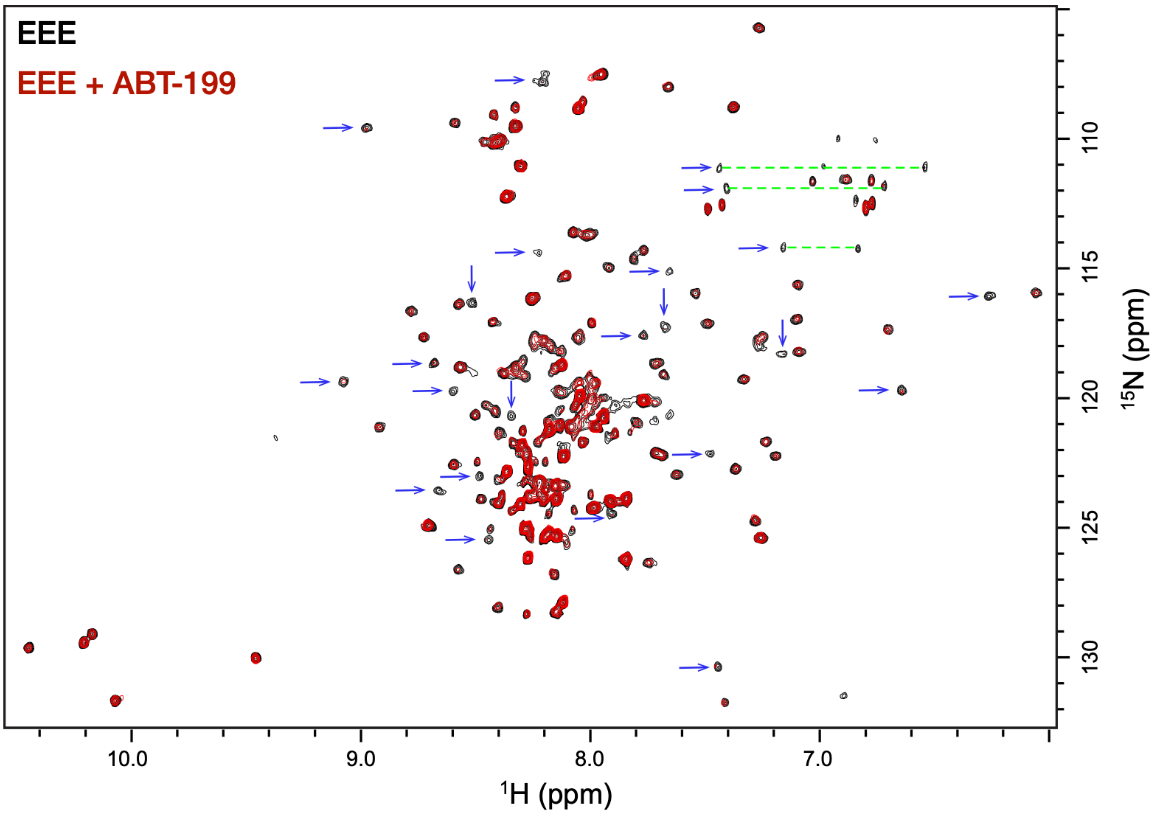

Supplementary Fig. 6: NMR study of EEE. The overlaid two-dimensional (2D) ^{1}H-^{15}N TROSY-HSQC spectra acquired from EEE (100 μM) in the absence (black) and presence (red) of ABT199 (200 μM). The amide resonances that exhibit the significant reduction in intensity after the addition of ABT199 are indicated with blue arrows. The side chain signals of Asn and Gln are connected with green dashed lines. The results provide additional evidence that the phosphomiemic mutant EEE reduces the structural flexibility of BCL-2 and it does bind to ABT-199.
