# Supplementary material for: Anti-apoptotic BCL-2 regulation by changes in dynamics of its long unstructured loop
Source: Commun Biol. 2020 Nov 12;3:668. doi: 10.1038/s42003-020-01390-6 (PMC7665024; doi:10.1038/s42003-020-01390-6)
Supplement: Supplementary file 2 — Description of Additional Supplementary Files [file 42003_2020_1390_MOESM2_ESM.pdf]

Description of Additional Supplementary Items

File Name: Supplementary Data

Description: Source data for the main figures
